# Paradigm shift in acute dizziness: is caloric testing obsolete?

**DOI:** 10.1007/s00415-021-10667-7

**Published:** 2021-06-30

**Authors:** Miranda Morrison, Athanasia Korda, Ewa Zamaro, Franca Wagner, Marco D. Caversaccio, Thomas C. Sauter, Roger Kalla, Georgios Mantokoudis

**Affiliations:** 1 Department of Otorhinolaryngology, Head and Neck Surgery, Inselspital, University Hospital Bern and University of Bern, 3010 Bern, Switzerland; 2 University Institute of Diagnostic and Interventional Neuroradiology, Inselspital, University Hospital Bern and University of Bern, Bern, Switzerland; 3 Department of Emergency Medicine, Inselspital, University Hospital Bern and University of Bern, Bern, Switzerland; 4 Department of Neurology, Inselspital, University Hospital Bern and University of Bern, Bern, Switzerland

**Keywords:** Caloric testing, Head-impulse test, Neuritis, Acute stroke, Vertigo, Dizziness

## Abstract

**Objective:**

Cold and warm water ear irrigation, also known as bithermal caloric testing, has been considered for over 100 years the ‘Gold Standard’ for the detection of peripheral vestibular hypofunction. Its discovery was awarded a Nobel Prize. We aimed to investigate the diagnostic accuracy of Caloric Testing when compared to the video head impulse test (vHIT) in differentiating between vestibular neuritis and vestibular strokes in acute dizziness.

**Design:**

Prospective cross-sectional study (convenience sample).

**Setting:**

All patients presenting with signs of an acute vestibular syndrome at the emergency department of a tertiary referral center.

**Participants:**

One thousand, six hundred seventy-seven patients were screened between February 2015 and May 2020 for Acute Vestibular Syndrome (AVS), of which 152 met the inclusion criteria and were enrolled. Inclusion criteria consisted of a state of continuous dizziness, associated with nausea or vomiting, head-motion intolerance, new gait or balance disturbance and nystagmus. Patients were excluded if they were younger than 18 years, if symptoms lasted < 24 h or if the index ED visit was > 72 h after symptom onset. Of the 152 included patients 85 completed testing. We assessed 58 vestibular neuritis and 27 stroke patients.

**Main outcome measures:**

All patients underwent calorics and vHIT followed by a delayed MRI which served as a gold standard for vestibular stroke confirmation.

**Results:**

The overall sensitivity and specificity for detecting stroke with a caloric asymmetry cut-off of 30.9% was 75% and 86.8%, respectively [negative likelihood ratio (NLR) 0.29] compared to 91.7% and 88.7% for vHIT (NLR 0.094). Best VOR gain cut-off was 0.685. Twenty-five percent of vestibular strokes were misclassified by calorics, 8% by vHIT.

**Conclusions:**

Caloric testing proved to be less accurate than vHIT in discriminating stroke from vestibular neuritis in acute dizziness. Contrary to classic teaching, asymmetric caloric responses can also occur with vestibular strokes and might put the patient at risk for misdiagnosis. We, therefore, recommend to abandon caloric testing in current practice and to replace it with vHIT in the acute setting. Caloric testing has still its place as a diagnostic tool in an outpatient setting.

**Supplementary Information:**

The online version contains supplementary material available at 10.1007/s00415-021-10667-7.

## Introduction

Since its discovery in 1907 by Robert Bárány [1], who received the Nobel prize in 1916 [2], Caloric Testing has been widely accepted as a Gold Standard for detecting a vestibular hypofunction, thus allowing the confirmation of peripheral vestibular disease in patients suffering from acute dizziness. However, the accuracy of calorics in discriminating vestibular neuritis from vestibular strokes in patients with acute dizziness is not known. One study reported a false negative rate of up to 22% of vestibular strokes [3]. Between 5 and 25% of isolated dizziness end up with a final diagnosis of posterior fossa infarction [3–5] with a reported initial misdiagnosis rate of up to 28% [6]. This is because central vestibular disorders mimic in many cases peripheral disease[7] also known as pseudo-neuritis. Fifty percent of dizzy patients with a stroke do not demonstrate any focal neurological signs [8]. Thermal (caloric) water irrigation of both ears stimulate the vestibular organ, particularly the horizontal semicircular canal, elicitating eye movements (nystagmus), which can be recorded with video Frenzels. This test, however, is very uncomfortable, consumes vast emergency department (ED) resources and is potentially less accurate due to great inter-subject and test–retest variability[9]. In view of all these disadvantages, any solution to avoid undertaking calorics and to increase diagnostic accuracy is crucial. Developed by Halmagyi and Curthoys [10], the Head Impulse Test (HIT) has proved itself as one of the most accurate triage tests in detecting vestibular strokes [3, 11]: Physicians have to move the patient’s head from side to side rapidly (impulse) inducing the vestibulo-ocular reflex (VOR). Head and eye movements can be recorded quantitatively with VOG goggles (video-HIT, or vHIT) [12, 13]. Disconcordant eye and head movements (pathologic VOR) indicate a peripheral vestibular deficit, such as vestibular neuritis. An intact VOR (concordant eye/head movements) would, however, be indicative of vestibular stroke. With the advent of digital technologies such as eye- and head-tracking by video-oculography (VOG) [11], it has been possible to offer non-invasive, time- and cost-efficient diagnostic techniques in the ED. Although many studies have investigated the correlation between caloric testing and vHIT, none have focused on acute vestibular disorders [14, 15]. We hyphothesized that a pathologic caloric exam without any additional test such as the vHIT might give ED physicians a false sense of security and put stroke patients at risk being misdiagnosed as vestibular neuritis. Unfortunately, only a small proportion of physicians perform systematically HITs in the ED [16].

In our study, we aim to investigate the diagnostic accuracy of Caloric Testing when compared to vHIT in differentiating between vestibular neuritis and vestibular strokes in acute dizziness.

## Materials and methods

**Fig. 1:**
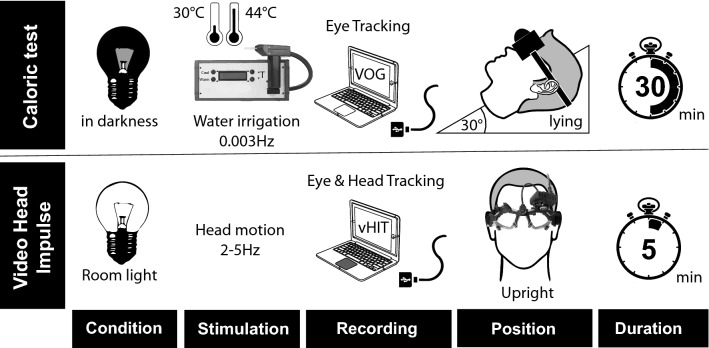
Technical setup for the caloric exam compared to the Video-Head Impulse test. Diagram comparing the technical setup for the caloric exam with that of the vHIT; calorics are performed in the dark on a patient in a supine position and head rest positioned at 30° from horizontal. The outer ear canal on each side is irrigated sequentially for 30 s (at 30° C cold and 44° C warm water) and the resulting eye movements recorded for a duration of 3 min using VOG goggles. The whole procedures takes up to 30 min including waiting intervals of 5 min between irrigations. The vHIT is performed in a normal lit room on a upright sitting patient. The head is moved rapidly from side to side (20 times in an impulse-like motion) and eye movements are recorded using adapted vHIT-goggles. When done correctly, the vHIT takes under 5 min

We conducted a prospective cross-sectional study (convenience sample), between February 2015 and May 2020, of all cases presenting with acute dizziness at the ED in a tertiary referral center. 1677 patients were screened for Acute Vestibular Syndrome (AVS) as part of a large cross-sectional study (DETECT—[Dizziness Evaluation Tool for Emergent Clinical Triage]), of which 152 met the inclusion criteria and were enrolled. Inclusion criteria consisted of a state of continuous dizziness, associated with nausea or vomiting, head-motion intolerance, new gait or balance disturbance and nystagmus. Patients were excluded if they were younger than 18 years, if symptoms lasted < 24 h or if the index ED visit was > 72 h after symptom onset. Figure 1S (Appendix) shows a flow diagram with all screened patients, inclusions and exclusions of dizzy patients. All enrolled patients underwent when feasible a thorough physical examination, Caloric Testing and vHIT testing. All patients received an MRI either at the index visit or a second, delayed MRI if there was no acute MRI indicated based on clinical grounds or if the first MRI was non-diagnostic. The delayed MRI served as a reference standard for stroke detection. Enrolled patients were clinically re-evaluated between day 3 and day 10, at day 30 and day 90. All images were reviewed by a certified second blinded neuroradiologist, discrepancies were resolved by consensus and inter-rater concordance reported. Figure 1 shows the two investigated tests, the required equipment, stimulation modalities and recording setup.

**Fig. 2:**
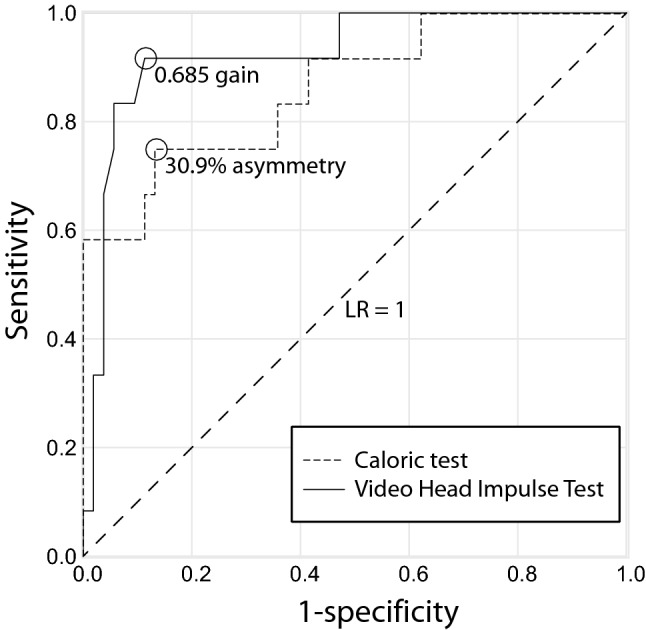
ROC curves. ROC curve demonstrating a higher sensitivity and specificity for vHIT for the detection of stroke compared to calorics. Black circles indicate the optimal test discrimination cut-off for each test. The dotted line illustrates a likelihood ratio of 1 with an area under the curve (AUC) at 0.5 indicating an unhelpful test

We performed caloric tests irrigating sequentially both ears with warm (44 °C) and cold (30 °C) water for 30 s and a total water volume of 250 ml (Vario Otopront device) in patients lying 30° supine (Fig. 2S, supplementum, panel A). Intervals between irrigations were 5 min long, starting first with warm irrigation on the right ear. Convection flows of inner ear fluid (particularly in the horizontal semicircular canal) produced horizontal eye movement responses (nystagmus), which were recorded in darkness (blocked visual fixation) with a calibrated VOG device (EyeSeeCam, Munich). The Cut-off for pathologic Caloric responses was 20% asymmetry [17], which was calculated using Jongkee’s formula [18] after correcting for spontaneous nystagmus.

In contrast, vHIT was performed solely on the lateral canal by fast passive horizontal head movements (high frequency, 10–20° head excursion in 100–300 ms corresponding to a 1000–6000°/sec^2^ acceleration) in room light during visual target fixation at > 1 m distance. We recorded head and eye movement velocity with a head mounted infrared highspeed camera (EyeSeeCam, Munich) connected to a laptop by USB (Fig. 2S, Panel B). VOR gain values were derived from eye velocity divided by head velocity at 60 ms after HIT onset [19]. vHIT exams were classified as abnormal based on VOR gains (Gain < 0.79 based on own laboratory normative values) and the presence of corrective saccades. Additionally we collected information on age, gender, duration of symptoms, and other associated relevant otological or neurological symptoms.

### Statistics

Cohen’s Kappa was calculated for the assessment of inter-rater agreement between two experienced neuroradiologist. Descriptive statistics were reported using SPSS statistical software (IBM SPSS Statistics for Windows, Version 25.0. Armonk, NY: IBM Corp.). We used a binary logistic regression to evaluate stroke predictors derived from caloric and vHIT exams in 65 patients who underwent both test modalities. We calculated a receiver characteristics curve (ROC) with its corresponding sensitivity, specificity, accuracy and negative likelihood ratio with its impact on post-test probability for each test. Best cut-off points based on Youden’s J. We followed the STARD guidelines for reporting the diagnostic accuracy. Our estimated sample size was 52 with an estimated marginal error of 0.1 (95% CI 80% power) and a diagnostic accuracy (AUC) of 0.90 for vHIT. The two ROC curves were compared using the method of DeLong et al. [20].

## Results

We screened 1677 patients with AVS of which 152 patients were enrolled aged between 20 and 91 (mean 55.67 years). Out of 152 patients, 58 were diagnosed with vestibular neuritis (mean age 54 years ± 15.7), while the remaining 27 patients were vestibular strokes (mean age 62.1 years ± 15.9 years). Vascular territories included the PICA (17), SCA (3), AICA (2), basilar artery (3), vertebral artery (2), anterior (1) and middle cerebral artery (4). There was an excellent inter-rater agreement regarding masked MRI assessment (94%, *κ* = 0.78). None of the patients with a normal caloric response had an abnormal vHIT (Table 1). Patients with an abnormal vHIT, however, systematically showed a pathologic caloric response as well. Table 1 shows the number of concordant or disconcordant exams comparing vHIT with calorics. Every increase of 0.1 VOR gain increased significantly the stroke risk (OR 2.832, 95% CI 1.5–5.2, *P* < 0.001, Table 2). A decreased asymmetry of 1% steps, however, decreased slightly the stroke risk (OR 0.926, 95% CI 0.88–0.97, *P* = 0.001, Table 2). Figure 2 shows the receiver operating characteristic (ROC) curves for vHIT (AUC = 0.93, 95% CI 0.84–1.00, *P* < 0.001) and calorics (AUC = 0.86, 95% CI 0.74–0.99, *P* < 0.001) with curves going to the left upper corner. There was no statistical difference between the two ROC curves (*P* = 0.22).

**Table 1:**
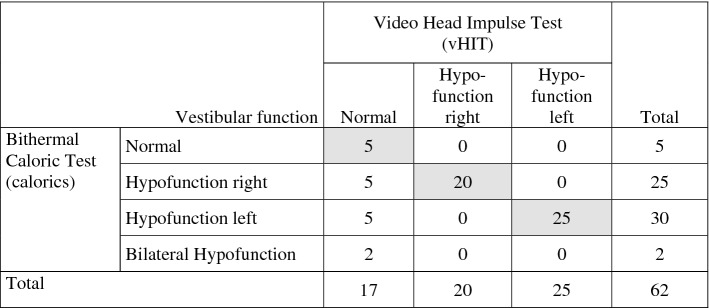
Concordance vHIT versus calorics

**Table 2:** Stroke risk estimation for vHIT and caloric asymmetry

	Increment steps	Regression coefficient	Standard error	Wald	*df*	*P* Value	Odd's ratio	95% Confidence interval	
								Lower	Upper
vHIT gain	0.1	1.041	0.308	11.387	1	0.001	2.832	1.547	5.183
Caloric asymmetry	1%	− 0.077	0.024	10.446	1	0.001	0.926	0.883	0.970

The overall sensitivity in discriminating strokes with caloric testing was 75% with a specificity of 86.8% (Table 3). The accuracy of caloric testing was 84.6% using a cut-off of 30.9% asymmetry. The accuracy of vHIT in detecting stroke was 89.3% with a sensitivity of 91.7% and specificity of 88.7% using a cut-off of 0.685 VOR gain. Table 3 shows alternative cut-off values and their corresponding sensitivity/specificity. The negative likelihood ratio for ruling-out stroke was 0.288 for calorics and 0.094 for vHIT (Table 3). Table 4 shows the pre-test and post-test probabilities of stroke assuming pre-test probabilities based on risk stratification rules. Table 4 illustrates the impact of the negative likelihood ratio (NLR) on stroke probability. Stroke probability decreased by 9–53% points after a vHIT exam and by 6.9–28.6% points after calorics depending on the assumed pre-test probability.

**Table 3:** Sensitivity and specificity for vHIT and calorics

	vHIT(Gain)		Calorics (% Asymmetry)	
Test cut-off	> 0.685	> 0.805	< 25.3%	< 30.9%
AUC (95% CI)	0.926 (0.833–0.976)		0.863 (0.755–0.936)	
Sensitivity	91.7%	41.7%	58.3%	75%
Specificity	88.7%	96.2%	96.2%	86.8%
Negative test	48	58	56	49
Positive test	17	7	9	16
True positives	11	5	7	9
False positives	6	2	2	7
True negatives	47	51	51	46
False negatives	1	7	5	3
Likelihood ratio pos. test	8.097	11.042	15.458	5.679
Likelihood ratio neg. test	0.094	0.606	0.433	0.288
Accuracy	89.3	86.2	89.2	84.6

**Table 4:** Pre-test and post-test probabilities of stroke using calorics or vHIT to ‘rule out’ stroke

	Post-test probability of stroke	
Test	Calorics (rule out stroke)	vHIT (rule out stroke)
Test cut-off	30.9% asymmetry	0.685 gain
Pre-test probability of stroke (based on risk stratification rules)	Sn 75%, Sp 86.8% NLR 0.29	Sn 91.7%, Sp 88.7% NLR 0.094
10% (low)	3.1%	1.0%
25% (average)	8.8%	3.0%
50% (high)	22.4%	8.6%
75% (very high)	46.4%	22.0%

*Sn* sensitivity, *SP* specificity, *NLR* negative likelihood ratio

## Discussion

The presence of caloric asymmetry is used in the emergency department as a means of confirming neuritis in patients with AVS and complete absence of neurological symptoms. If an asymmetrical caloric test was synonymous of pathological peripheral function (neuritis), every fourth vestibular stroke would have been missed. We confirmed our hypothesis that a pathological caloric test without additional clinical tests such as the bedside vHIT puts a stroke patient at risk being misclassified as a vestibular neuritis. Considering currently accepted test cut-off values for caloric response asymmetry, even every 2^nd^ to 3^rd^ patient would have been misdiagnosed. On the contrary, a vHIT test yields a significantly higher sensitivity and specificity for vestibular stroke detection. An increased gain value was significantly associated with stroke. A pathological vHIT result with low gain was, however, systematically associated with a pathological Caloric Test result. We never observed a normal caloric test when vHIT was abnormal and thus, there is no need to perform any caloric testing to confirm a pathologic vHIT.

### Is caloric testing accurate for discrimination between peripheral and central dizziness?

Admittedly, for calorics, we observed a decent decrease of stroke risk for every 1% of increase in asymmetry, which was not described in the literature before. The cut-off value, however, to rule-out stroke was higher (31% asymmetry) compared to the test cut-off used in laboratories (20–25%) for the detection of vestibular deficits [17]. We demonstrated, that central lesions can cause pathologic caloric responses in every 4th patient, which is in line with the current literature[3] (supplemental material). Thus, a pathologic caloric response is not a hallmark for a peripheral lesion but rather documents a deficit of vestibular pathways at any neuronal level in the low frequency range.

### vHIT is more sensitive for vestibular stroke detection

Each incremental increase in VOR gain in vHIT resulted, however, in a significant incremental increase in stroke risk. Our test cut-off of 0.685 gain confirmed previously reported test thresholds for stroke discrimination with vHIT [21]. Based on current literature, we recommend, however, to assess additionally the gain asymmetry and the corrective saccade amplitude in order to further increase the accuracy of vHIT in detecting stroke [22]. Even clinically performed HITs (which assess VOR function qualitatively by simple eye observation looking for corrective saccades) yield a high sensitivity of stroke detection in acute dizziness, provided that they are performed by experts. However, a quantitative method, such as vHIT, would allow for more reliable and more examiner independent results. A recent study from our group showed, that even non-experts and novices were able to perform valid HITs using a video recording system [23]. Thus, a point-of care examination with vHIT in the ED has the potential of a widespread use, providing an accurate, cost-efficient and non-invasive method for stroke detection in acute dizzy patients.

### Can the caloric test be replaced by the vHIT in the acute setting?

vHIT demonstrated a better accuracy with a higher sensitivity/specificity in the detection of vestibular strokes. The rate of missed patients (false negative result) having a serious cause of dizziness was significantly lower with vHIT. The false positive rate, however, was the same for both tests. In a previous cross-sectional study, vHIT was found to be even more sensitive for stroke detection than MRI [21, 24] [21], if performed within 24 h [3]. This finding was confirmed by others [25, 26]. There are no publications investigating or comparing the accuracy of vHIT versus calorics in detecting vestibular strokes in patients with an acute vestibular syndrome. Both exams have been extensively compared regarding the detection of vestibular deficits in sub-acute or chronic stages [14, 15], however, such deficits might originate from peripheral or central causes. Rather than seeking to initially detect a benign vestibular deficit with calorics, emergency physicians and general practitioners should prioritise the exclusion first and foremost of any dangerous cause of acute dizziness with vHIT. Benign causes of dizziness could be further assessed and treated as a second line, for example in an outpatient, sub-acute setting. We therefore suggest a paradigm shift towards modern vHIT testing in patients with acute dizziness and to abandon calorics in the acute setting.

### When to perform calorics

There are differences reported in the literature regarding the detection of vestibular deficits [14, 15], not specifically vestibular strokes. Comparing vHITs to calorics is like comparing apples to pears [27]; Calorics represent the measurement of low frequency stimulation of horizontal semicircular canals only, whereas vHITs concentrate of high frequencies and test all six canals in all spatial planes. It is, therefore, not surprising to find a dissociation of the two.

Other articles did compare the clinical HIT (before the advent of video-oculography) with calorics [28]. These articles support the idea that there is a low correlation between both exams, the vHIT often having a very low sensitivity for the detection of canal paresis [14, 15]. A dissociation with abnormal calorics and normal vHIT can also be seen in patients with a mild vestibular hypofunction if this canal paresis exceeds ~ 40% caloric asymmetry [28–30]. vHIT sensitivity in detecting acute vestibular deficits is higher (63%) and lower in chronic dizziness (33%) [31] with an overall reported sensitivity ranging from 41 to 86% [25]. Caloric tests, however, are more sensitive to diagnose Menière’s disease [32] being often abnormal while vHIT is not affected. Calorics measure, however, only one single semicircular canal function and might miss an incomplete neuritis (inferior neuritis) [33].

Because the range calorics measure differ from vHIT (low frequency vs high frequency), it should not completely be excluded altogether. Rather, it should be seen as a complementary exam of vestibular function. Thus, patients presenting with a clinical picture of AVS, with a normal delayed MRI (3–10 days after symptom onset) and a normal vHIT, would be good candidates for further investigations by Calorics. If this is the case, one could argue for the redundancy of caloric testing in the acute setting and argue for its relegation to a later phase of non-acute testing to extend investigations of vestibular function. However, there is no need to perform calorics if vHIT is abnormal, since we never observed a dissociation of the two tests when vHIT was abnormal.

### Strength and limitations

Our paper is the first large study offering a direct comparison of Caloric Testing and vHIT in acute vestibular syndrome, however, our results are not generalizable to all dizzy patients. In addition, we did not assess the absolute canal function or the sum of the peak slow phase velocity of caloric nystagmus, since absolute values would not be generalizable due to the absence of any normalization procedure. Reporting the asymmetry of canal function alone is, however, not meaningful in patients with a bilateral vestibulopathy or presbyvestibulopathy. In addition, there is large variability regarding the thermal energy applied to the lateral canals compared to standardized horizontal head movements in vHIT.

Careful vHIT interpretation is advised in patients with other pathologies such as e.g. Menière’s Disease, vestibulare Schwannoma [25], vestibular migraine or BPPV, which can cause episodic dizziness and thus, bearing the risk of finding an asymptomatic patient at the examination time point. Best sensitivity of vHIT is yielded in patients having continuous dizziness associated with spontaneous nystagmus, which serves as an objective clinical sign of dizziness and underlying severity of vestibular imbalance. We also had a larger number of vHIT results versus caloric results; this could be due to a refusal by highly symptomatic patients to undergo caloric investigation. This could potentially lead to a selection bias in that highly symptomatic patients where inadvertently excluded from the study.

### Clinical implication

Our study results have an immediate impact in current clinical practice suggesting a paradigm shift from calorics towards a modern vHIT exam. Caloric testing has a variety of limitations: (1) only one semicircular canal (horizontal) is stimulated per ear while the remaining four canals remain unassessed, (2) the stimulus is non-physiological stimulating at low frequencies only (< 0.003 Hz) and non-reciprocal (stimulus from the contralateral ear missing), (3) it is very disagreeable for patients, inducing vertigo lasting up to several minutes, (4) there is a large inter-subject variability due to ear anatomy resulting in a variable application of thermal energy, (5) it needs a special and stationary irrigation device to maintain a constant water temperature with a purified water supply, (6) has to be performed in total darkness (adapted room) with Frenzel or video Frenzel goggles to remove visual fixation and finally, (7) it is costly consuming both, vast human and time resources. We, therefore, suggest to replace calorics with a more convenient and simple vHIT in AVS patients in view of its non-inferiority.

## Conclusions

Caloric testing proved to be less accurate than vHIT in discriminating neuritis from vestibular stroke in acute dizziness. Contrary to classic teaching, asymmetric caloric responses can also occur with vestibular strokes and might put the patient at risk for misdiagnosis. We, therefore, recommend to abandon caloric testing in current practice. vHIT could serve as a replacement test in the acute setting. Caloric testing has still its place as a diagnostic tool in an outpatient setting.

## Data sharing statement

The data that support the findings of this study are available from the corresponding author, [GM], upon reasonable request.

## Supplementary Information

Below is the link to the electronic supplementary material.

Supplementary file1 (PDF 594 KB)

## Data Availability

The lead author affirms that this manuscript is an honest, accurate, and transparent account of the study being reported; that no important aspects of the study have been omitted; and that any discrepancies from the study as planned (and, if relevant, registered) have been explained.

## Declarations

### Dissemination to participants and related patient and public communities

There are no plans to disseminate the results of the research to study participants or the relevant patient community.
