# Supplementary material for: Paradigm shift in acute dizziness: is caloric testing obsolete?
Source: J Neurol. 2021 Jun 30;269(2):853–60. doi: 10.1007/s00415-021-10667-7 (PMC8782777; doi:10.1007/s00415-021-10667-7)
Supplement: Supplementary file 1 — Supplementary file1 (PDF 594 KB) [file 415_2021_10667_MOESM1_ESM.pdf]

Supplementary Appendix

Table of contents

Figure 1S, Flow Chart	Page 2
Figure 2S, Examination Setup	Page 3

Figure 1S. Flow chart: showing the screening and enrollement process.

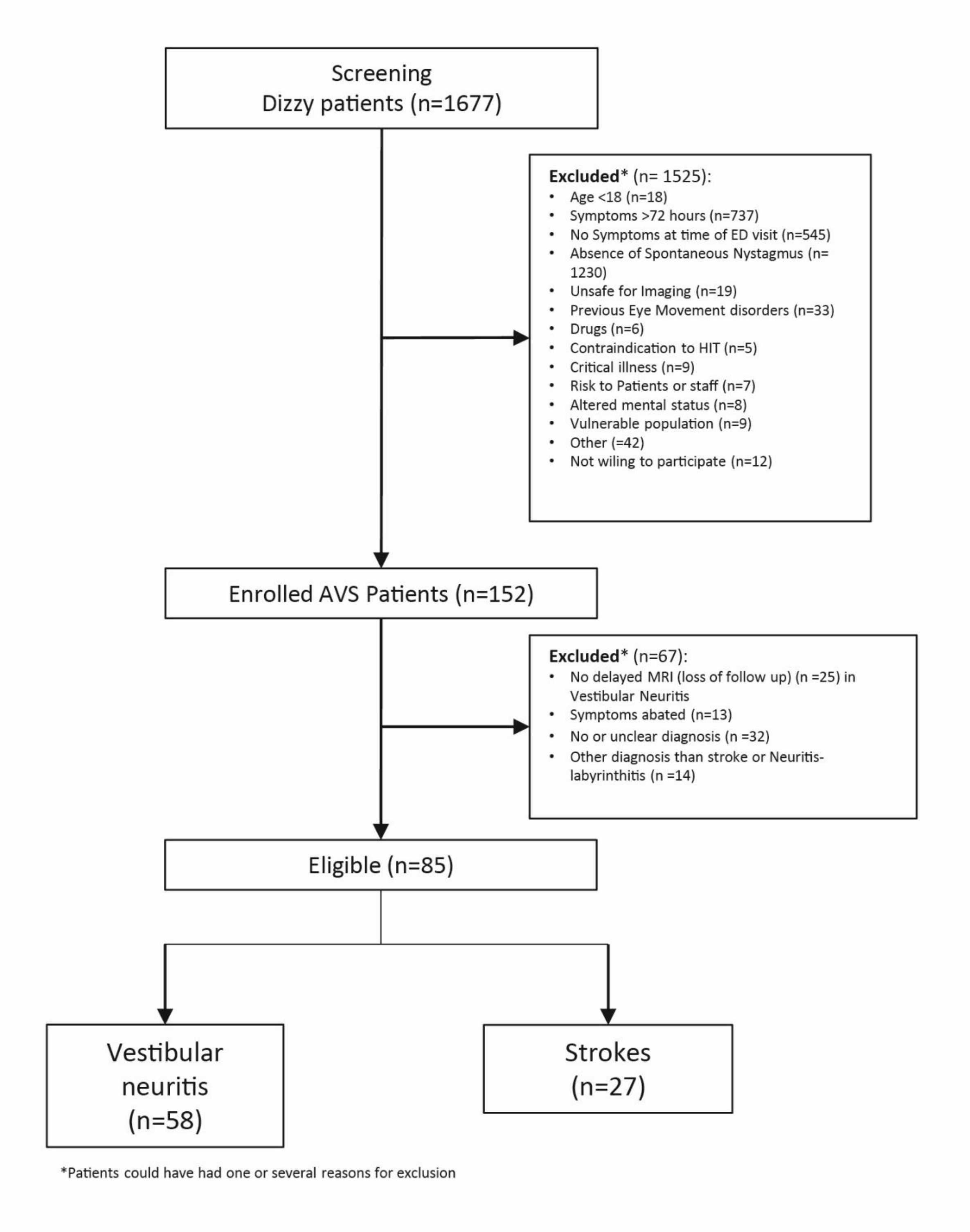

Figure 2S. Examination setup

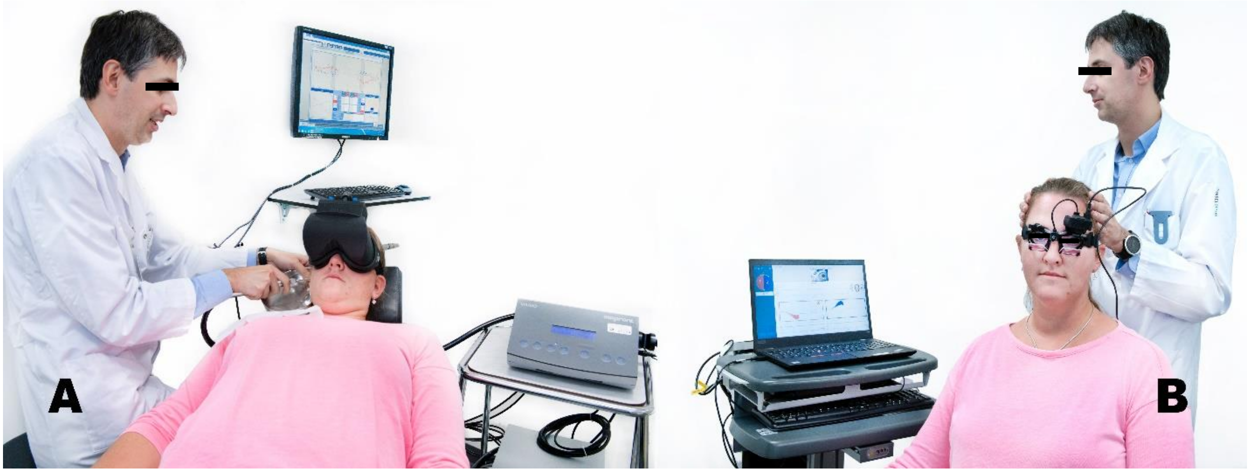

Figure 2S. Illustration of caloric ear irrigation (A) using water at 7°C below and 7°C above body temperature for 30 seconds. Eye movement responses (slow phase velocity of nystagmus) are recorded with VOG for 3 minutes. The video head impulse test (vHIT) (B) consists of rapid passive head movements towards each side. The patient fixates a target point >1m distance. Head and eye velocity are tracked and recorded during movements using light-weight vHIT goggles with a build-in accelerometer and high speed infrared camera connected to a laptop.
